# Implementing an Advanced Thoracic Surgery Program in Rural Haiti: Challenges of High Complexity Surgery in a Low Income Country

**DOI:** 10.1186/1749-8090-10-S1-A19

**Published:** 2015-12-16

**Authors:** Pierre Theodore, Deborah Jenny Robert, Alexis N Bowder, Mac Lee Jean Louis, Luther Ward

**Affiliations:** 1 Department of Surgery, UCSF, San Francisco, CA, 94143, USA; 2 General Surgery, Hôpital Universitaire de Mirebalais, Mirebalais, Haiti; 3 Program in Global Surgery and Social Change, Harvard Medical School, Boston, MA, 02115, USA; 4 University of Nebraska School of Medicine, Omaha, NE, 68106 USA; 5 Partners in Health, Boston, MA, 02215, USA

## Background/Introduction

Recently, the Lancet Commission on Global Surgery estimated that 5 billion people lack access to surgical care. While barriers of funding, training, staff and resources are substantial, demand for surgical care is immense in Low Income Countries. For the last two years the Hôpital Universitaire de Mirebalais (HUM) in Haiti has provided surgical care to over 4,000 patients. Recently a pilot trial of advanced thoracic surgery has been established.

## Aims/Objectives

As a demand for cardiothoracic surgical care becomes pressing, an increasing importance will be placed on the documentation of the successful implementation and the challenges of advanced surgical intervention in low-income settings. This is of particular importance in areas of endemic TB and iodine deficiency.

## Method

This is a retrospective review of 10 recent complex thoracic cases, including VATS, performed in a rural Haiti. Outcomes, length of stay complications, staffing requirements, infrastructural limitations were recorded through chart review and provider interviews.

## Results

10 patients are presented from the pilot program including massive mediastinal goiter with vascular involvement, 4 bronchopleural fistulae and empyema, chest wall sarcoma and reconstruction, flail chest with pulmonary contusion and 3 thoracotomies for trauma related hemorrhage. All non-emergent cases were presented for review through online portals with allied academic medical centers in the United States for diagnosis, pathology review, and collaborative planning of surgical procedures. Approaches included median sternotomy, video assisted thoracic surgery, and posterolateral thoracotomy. One perioperative death occurred in a multi-trauma patient but no elective procedures were associated with major adverse event. Intensive care monitoring, consistent single lung ventilation, blood bank issues, and airway management/bronchoscopy were infrastructural limitations noted.

## Discussion/Conclusion

While resources are inequitably distributed across the globe, burdens of surgical disease are great in low income countries. Proper patient selection, infrastructural support and acute care services are required for success. Informatic tools such as online collaboration, and telemedicine will likely aid in overcoming staffing and operative planning issues. With careful implementation complex thoracic procedures can be performed in low income settings with good perioperative outcomes

